# TFEB-driven lysosomal biogenesis is pivotal for PGC1**α**-dependent renal stress resistance

**DOI:** 10.1172/jci.insight.126749

**Published:** 2019-04-18

**Authors:** Matthew R. Lynch, Mei T. Tran, Kenneth M. Ralto, Zsuzsanna K. Zsengeller, Vinod Raman, Swati S. Bhasin, Nuo Sun, Xiuying Chen, Daniel Brown, Ilsa I. Rovira, Kensei Taguchi, Craig R. Brooks, Isaac E. Stillman, Manoj K. Bhasin, Toren Finkel, Samir M. Parikh

**Affiliations:** 1 Division of Nephrology,; 2 Department of Medicine, Beth Israel Deaconess Medical Center and Harvard Medical School, Boston, Massachusetts, USA.; 3 Center for Molecular Medicine, National Heart, Lung and Blood Institute, NIH, Bethesda, Maryland, USA.; 4 Department of Pathology, Beth Israel Deaconess Medical Center and Harvard Medical School, Boston, Massachusetts, USA.; 5 Division of Nephrology and Hypertension, Department of Medicine, Vanderbilt University Medical Center, Nashville, Tennessee, USA.; 6 Aging Institute of UPMC and the University of Pittsburgh, Pittsburgh, Pennsylvania, USA.

**Keywords:** Nephrology, Mitochondria

## Abstract

Because injured mitochondria can accelerate cell death through the elaboration of oxidative free radicals and other mediators, it is striking that proliferator γ coactivator 1-α (PGC1α), a stimulator of increased mitochondrial abundance, protects stressed renal cells instead of potentiating injury. Here, we report that PGC1α’s induction of lysosomes via transcription factor EB (TFEB) may be pivotal for kidney protection. CRISPR and stable gene transfer showed that PGC1α-KO tubular cells were sensitized to the genotoxic stressor cisplatin, whereas Tg cells were protected. The biosensor mitochondrial-targeted Keima (mtKeima) unexpectedly revealed that cisplatin blunts mitophagy both in cells and mice. PGC1α and its downstream mediator NAD^+^ counteracted this effect. PGC1α did not consistently affect known autophagy pathways modulated by cisplatin. Instead RNA sequencing identified coordinated regulation of lysosomal biogenesis via TFEB. This effector pathway was sufficiently important that inhibition of TFEB or lysosomes unveiled a striking harmful effect of excess PGC1α in cells and conditional mice. These results uncover an unexpected effect of cisplatin on mitophagy and PGC1α’s reliance on lysosomes for kidney protection. Finally, the data illuminate TFEB as a potentially novel target for renal tubular stress resistance.

## Abstract 2

PGC1α is renoprotective in the setting of platinum-based chemotherapy through the induction of mitophagy and lysosomal biogenesis via transcription factor EB.

## Graphical abstract

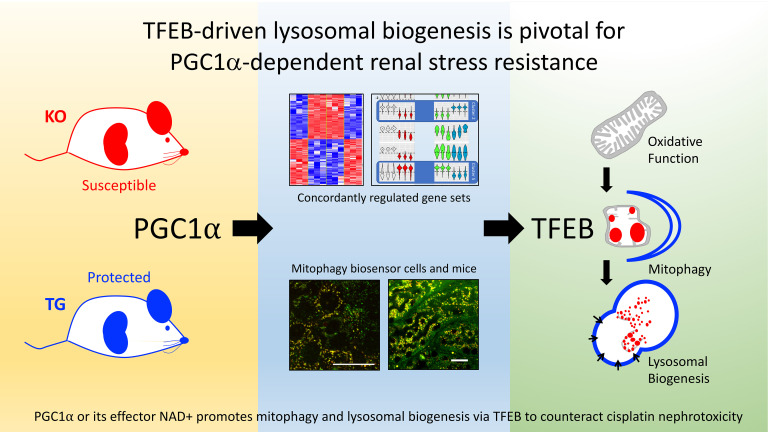

## Introduction

Mitochondria are critical for normal kidney function, evidenced by the high penetrance of tubulopathy among people with monogenic mitochondrial diseases (1). However, mitochondria also amplify injury responses to diverse stressors, including ischemia and inflammation (2–4). The injured mitochondrion promotes cell death by producing excessive free radicals and other proapoptotic mediators (4, 5). Thus, while mitochondria enable life-sustaining functions, they also accelerate cell death when stressed.

From this perspective, increased mitochondrial abundance prior to injury should favor cell death rather than cell protection. However, we recently found that tubular induction of the mitochondrial biogenesis factor peroxisome proliferator γ coactivator 1-α (PGC1α) increased mitochondrial mass and also protected from ischemic and inflammatory renal injury (6). Kang and colleagues similarly observed tubular PGC1α-dependent kidney protection in a model of crystal nephropathy (7). In the brain, PGC1α limits the damage that can arise from mitochondrial oxidants by inducing antioxidant enzymes (8). However, renal tubular PGC1α does not induce such enzymes (6), leaving unaddressed how mitochondrial biogenesis and mitochondrially targeted antioxidants can both be beneficial in the same models of acute kidney injury (AKI).

These studies were initiated to ask whether PGC1α-dependent renoprotection extends to toxic AKI, a heretofore unaddressed question, to our knowledge. A combination of potentially novel genetic cellular models, genetic mouse models, cellular and murine biosensor studies, and unbiased profiling yielded unanticipated insights regarding PGC1α-dependent mitochondrial quality control mechanisms in the renal tubule; our understanding of cisplatin nephrotoxicity; and transcription factor EB (TFEB) as a potentially new target for renal stress resistance.

## Results

**Figure 1:**
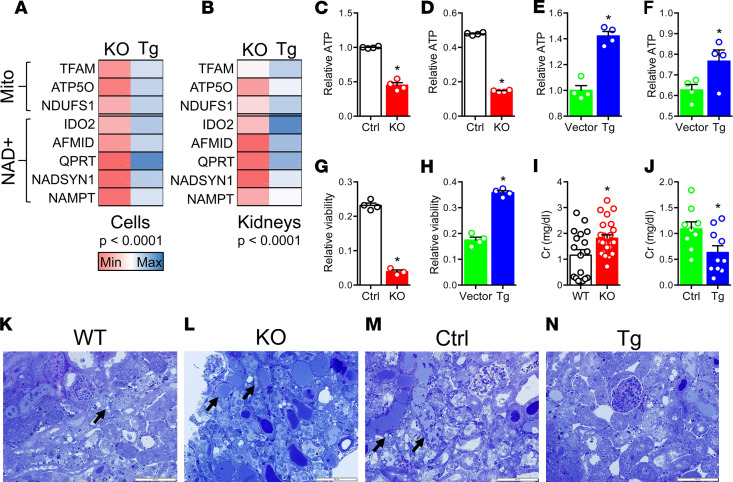
Loss or gain of PGC1α confers concordant effects against cisplatin toxicity in cells and in mice. (**A** and **B**) Heatmaps comparing mitochondrial and NAD^+^ biosynthetic enzyme expression patterns of PGC1α-KO vs. Tg renal tubular cells and renal expression in KO mice vs. iNephPGC1α (Tg) mice. Each cell represents average expression scaled to respective control (*n* = 3–6/group). Multiple comparison *P* values by ANOVA. Gene abbreviations defined in Methods. (**C** and **D**) ATP abundance in Ctrl and PGC1α-KO cells at baseline (**C**) and after cisplatin (**D**) (10 μM, 24 hours). Data in **D** normalized to Ctrl cells at baseline. (**E** and **F**) ATP abundance in vector and PGC1α Tg cells at baseline (**E**) and after cisplatin (**F**) (10 μM, 24 hours). Data in **F** normalized to vector cells at baseline. (**G** and **H**) Viability via XTT assay after cisplatin (10 μM, 24 hours). Results normalized to respective controls at baseline. *n* = 4/group for **C–H**. (**I** and **J**) Serum creatinine (Cr, mg/dl) in PGC1α-KO mice (**I**, *n* = 18 WT vs. 25 KO mice) or iNephPGC1α (**J**, *n* = 9 Ctrl vs. 10 Tg mice) 72 hours after cisplatin (20 mg/kg i.p. in PGC1α-KO and 30 mg/kg in iNephPGC1α) vs. respective controls. (**K–N**) Toluidine blue–stained plastic sections of renal cortex from (representative of 3–4 mice from **I** and **J**) with black arrows to necrotic tubules. Scale bars: 100 μm. *P < 0.05 by Mann-Whitney *U* test for **C–J**.

We applied CRISPR to crete stable PGC1α knockout renal tubular cells to study PGC1α by CRISPR (Supplemental Figure 1; supplemental material available online with this article; https://doi.org/10.1172/jci.insight.126749DS1) and stable PGC1α Tg counterparts by lentiviral gene transfer (Supplemental Figure 2). The cells exhibited expected changes in cellular respiration (Supplemental Figure 3). For both the canonical mitochondrial biogenesis function of PGC1α and the more recently described induction of NAD^+^ biosynthesis (6, 9), these cells paralleled kidney expression patterns of PGC1α-KO and Pax8-rtTA x tetO-PGC1α conditional tubular Tg (hereafter referred to as iNephPGC1α) mice (Figure 1, A and B). After completing concentration-ranging studies in cells (Supplemental Figure 4A), we found that KO cells produced baseline less ATP at baseline and suffered greater ATP depletion following cisplatin than control cells (Figure 1, C and D). Conversely, PGC1α Tg cells produced more baseline ATP and preserved ATP following cisplatin (Figure 1, E and F). Viability assays were analogous: KO cells were more susceptible to cisplatin, whereas Tg cells were more resistant (Figure 1, G and H). Given these concordant results, we tested different doses of cisplatin in mice (Supplemental Figure 4B) and then treated PGC1α-KO mice and iNephPGC1α mice with cisplatin. The former developed worse AKI, as assessed by the filtration marker serum creatinine, whereas the latter were more resistant to cisplatin than respective controls (Figure 1, I–N). Cisplatin-mediated injury to tubules was also quantified by the expression of kidney injury molecule-1 (KIM1) in renal homogenates from these experiments (Supplemental Figure 5). These studies confirmed the effects of PGC1α gene modulation observed on cisplatin cytotoxicity in vitro and nephrotoxicity in vivo. Together, these results establish the utility of KO and Tg cells as models of tubular PGC1α. They show for the first time to our knowledge that PGC1α is required for resistance to cisplatin nephrotoxicity. Furthermore, forced tubular PGC1α is sufficient to promote resistance against this stressor. Finally, the salutary action of renal tubular PGC1α is cell autonomous.

**Figure 2:**
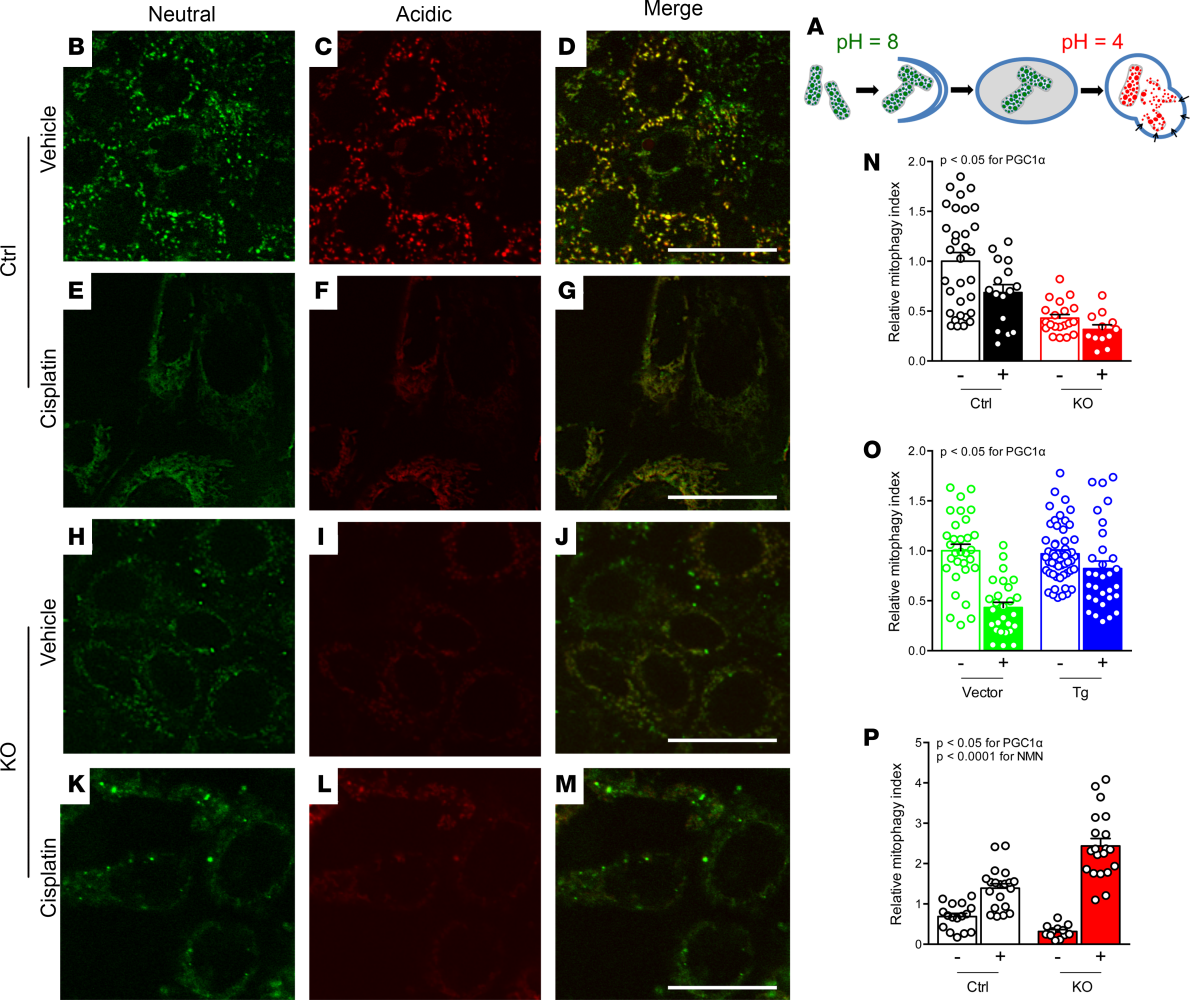
Cisplatin reduces mitophagy which PGC1α counteracts. (**A**) Color shift in mtKeima indicates mitophagy from green (neutral) to red (acidic). (**B–M**) PGC1α-KO cells or controls treated with vehicle or cisplatin (10 M, 24 hours). Scale bars: 5 μm. (**N**) Quantification of mitophagy index for **B–M** relative to control condition. *n* = 32, 16, 20, and 12 fields left to right from 3–4 biological replicates per condition. (**O**) Mitophagy index for PGC1α Tg cells or vector controls treated with vehicle or cisplatin (10 μM, 24 hours). *n* = 31, 27, 54, and 31 fields left to right from 3–6 biological replicates per condition. (**P**) PGC1α-KO cells treated with cisplatin (10 μM, 24 hours) ± concurrent nicotinamide mononucleotide (NMN, 1 mM). *n* = 16, 20, 12, and 20 fields left to right from 3–4 biological replicates per condition. Results analyzed by 2-way ANOVA on biological replicates with *P* values as indicated.

**Figure 3:**
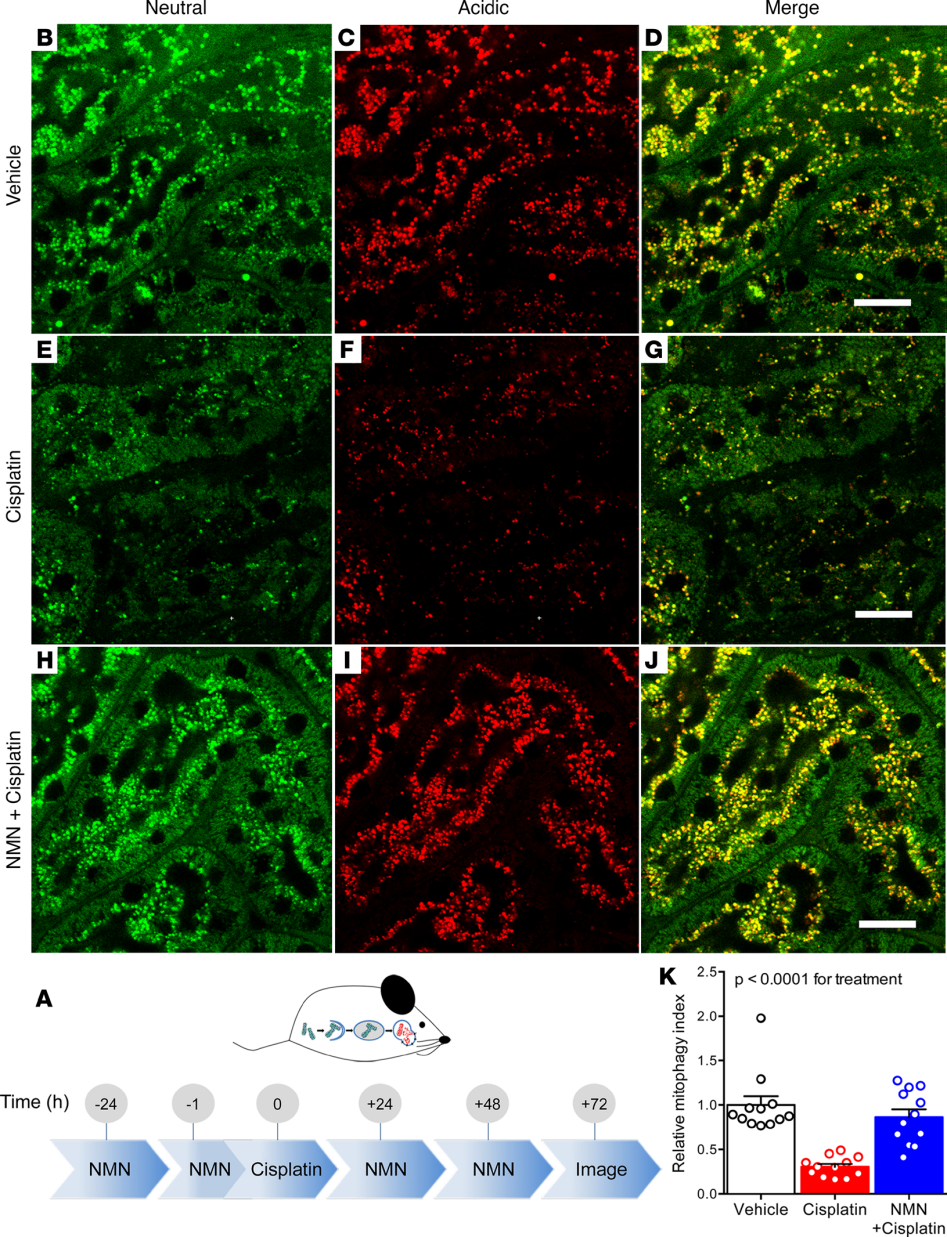
Cisplatin reduces mitophagy in vivo which NMN counteracts. (**A**) Schematic depicting experimental interventions in mtKeima Tg mice. **(29. AUTHOR: Query 28.)** (**B–J**) Renal cortex of mtKeima mice treated with vehicle or cisplatin (20 mg/kg i.p., 72 hours) ± NMN (400 mg/kg i.p. as indicated). Scale bars: 50 μm. (**K**) Quantification of mitophagy index for **B–J** relative to control condition. *n* = 12 fields from 3 mice per condition. *P* value calculated by ANOVA on biological replicates.

Earlier literature has reported that cisplatin augments autophagy (10, 11). Consistent with this concept, we found that levels of the autophagic marker p62 rose after cisplatin (Supplemental Figure 6). However, there was neither a robust nor concordant effect of PGC1α manipulation on p62. Cisplatin also increased PTEN-induced kinase protein 1 (PINK1), a mediator of mitophagy recently implicated in cisplatin toxicity (12–15). However, PGC1α did not exert a concordant effect on PINK1 in this setting (Supplemental Figure 7). To address further how cisplatin has been reported to induce these clearance mechanisms, while induction of such mechanisms also counteracts toxicity (16, 17), we next visualized mitophagy by stably expressing the biosensor mitochondrial-targeted Keima (mtKeima) in PGC1α-KO and Tg cells (18–20). This dual-fluorescent probe reports the pH shift as basic mitochondria undergo mitophagy in acidic lysosomes (Figure 2A). In contrast to a recent report (14), the data with mtKeima suggest that cisplatin decreased mitophagy in renal tubular cells (Supplemental Figure 8). Furthermore, in PGC1α-KO cells, basal mitophagy was decreased and cisplatin exacerbated this (Figure 2, B–N). In PGC1α Tg cells, mitophagy was preserved despite cisplatin (Figure 2O). Biosensor studies, therefore, uncovered new effects both of cisplatin and renal tubular PGC1α. Given the concordant effects on metabolism, viability and mitophagy (e.g., cisplatin reduces, PGC1α depletion exacerbates, and PGC1α induction ameliorates), we tested augmentation of NAD^+^, an emerging mimetic of PGC1α’s renal effects with translational potential (6, 21). Application of the precursor nicotinamide mononucleotide (NMN) restored mitophagy in cisplatin-treated PGC1α-KO cells (Figure 2P). Tg mtKeima mice verified that cisplatin reduced renal tubular mitophagy, an effect that NMN counteracted (Figure 3).

**Figure 4:**
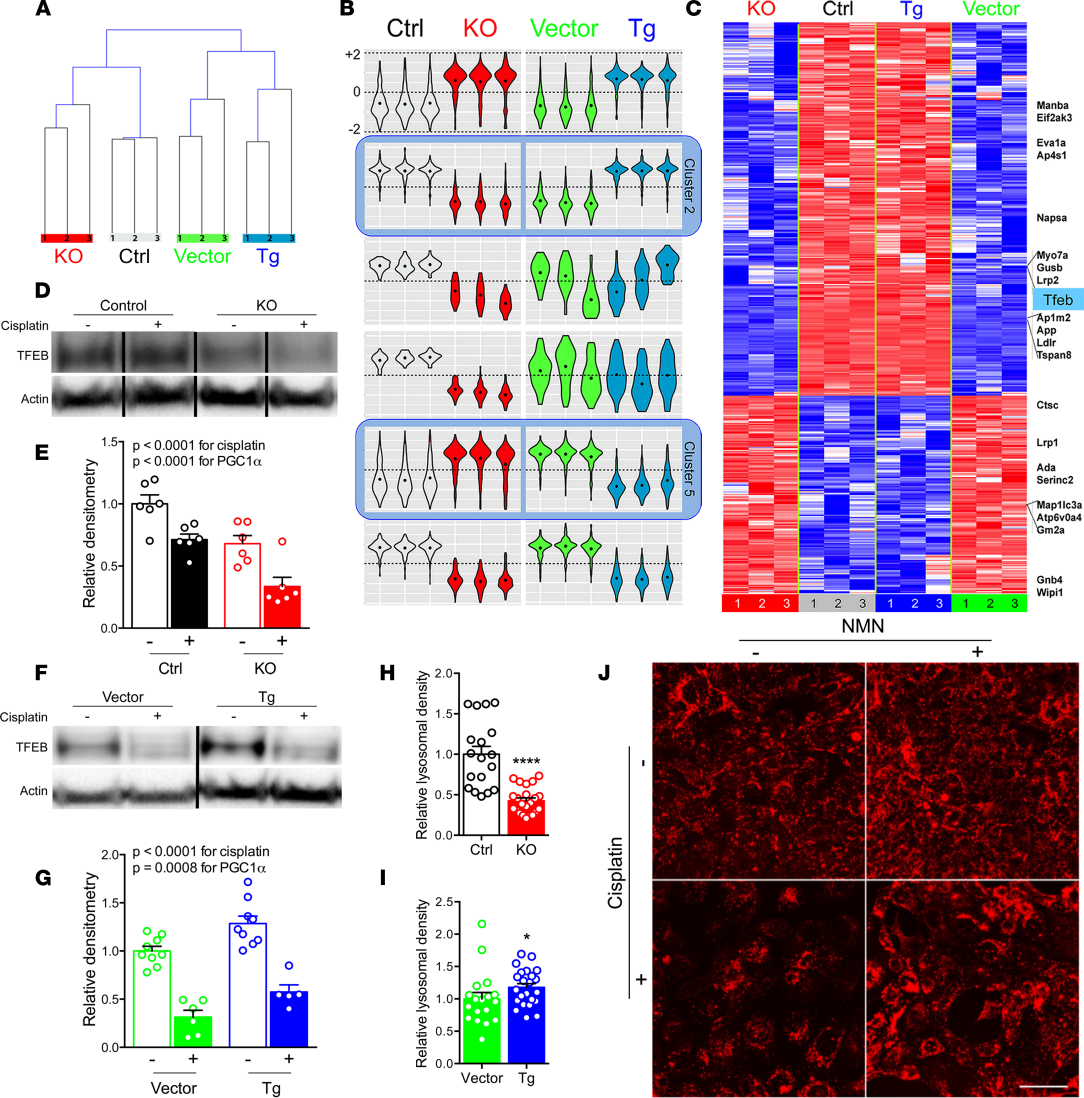
PGC1α promotes lysosomal biogenesis via TFEB. (**A**) Hierarchical clustering of cisplatin-treated (10 μM, 24 hours) PGC1α-KO cells or Tg cells and respective controls. (**B**) Gene expression clusters identified by self-organizing maps analysis. (**C**) Heatmap of transcripts in clusters 2 and 5 (i.e., oppositely regulated PGC1α transcripts). (**D–G**) Representative Western analysis and densitometry of TFEB in cisplatin-treated (10 μM, 12 hours) PGC1α-KO cells vs. controls. *n* = 6/condition (**D** and **E**); analogous for PGC1α Tg cells (**F** and **G**), *n* = 5–9/condition. *P* values as indicated by 2-way ANOVA. (**H** and **I**) Lysosomal abundance quantified from LysoTracker-stained PGC1α-KO (*n* = 18 control vs. 22 KO) or Tg cells (*n* = 18 vector vs. 23 Tg). (**J**) Representative LysoTracker images (from *n* = 18–24/condition) following cisplatin (10 μM, 24 hours) ± concurrent NMN (1 mM). Scale bar: 20 μm. *P < 0.05, ****P < 0.0001 by Mann-Whitney *U* test.

With unexpected evidence of (a) cisplatin reducing mitophagy and (b) PGC1α counteracting this effect without (c) impacting known cisplatin-dependent mechanisms, we conducted RNA sequencing to seek a mechanistic link by exploiting the symmetry of cisplatin’s effects in PGC1α-KO and Tg cells. Unsupervised hierarchical clustering confirmed intragroup homogeneity and intergroup distance between KO and Tg cells (Figure 4A). Supervised differential and self-organizing maps (SOM) analysis isolated transcripts whose expression was oppositely regulated by PGC1α-KO vs. Tg status (clusters 2 and 5 in Figure 4B). Among these was TFEB (Figure 4C), a master regulator of lysosomal biogenesis and autophagy (22). In neurons, PGC1α induces TFEB to counteract proteotoxicity (23). In nematodes, TFEB and PGC1α are mutually regulatory (24). Both a previously described TFEB-dependent gene set (25) and a curated database of genes involved in lysosomal biology (http://lysosome.unipg.it) were significantly overrepresented (*P* < 0.05) among PGC1α–oppositely regulated transcripts (Supplemental Figure 9). PGC1α-KO exacerbated, whereas PGC1α induction ameliorated, cisplatin’s suppressive effect on TFEB (Figure 4, D–G). Even absent cisplatin, lysosomal abundance mirrored PGC1α’s effects on TFEB; it was decreased in KO and increased in Tg cells relative to controls (Figure 4, H and I). When cisplatin was applied, lysosomal abundance decreased — an effect that NMN again counteracted (Figure 4J).

**Figure 5:**
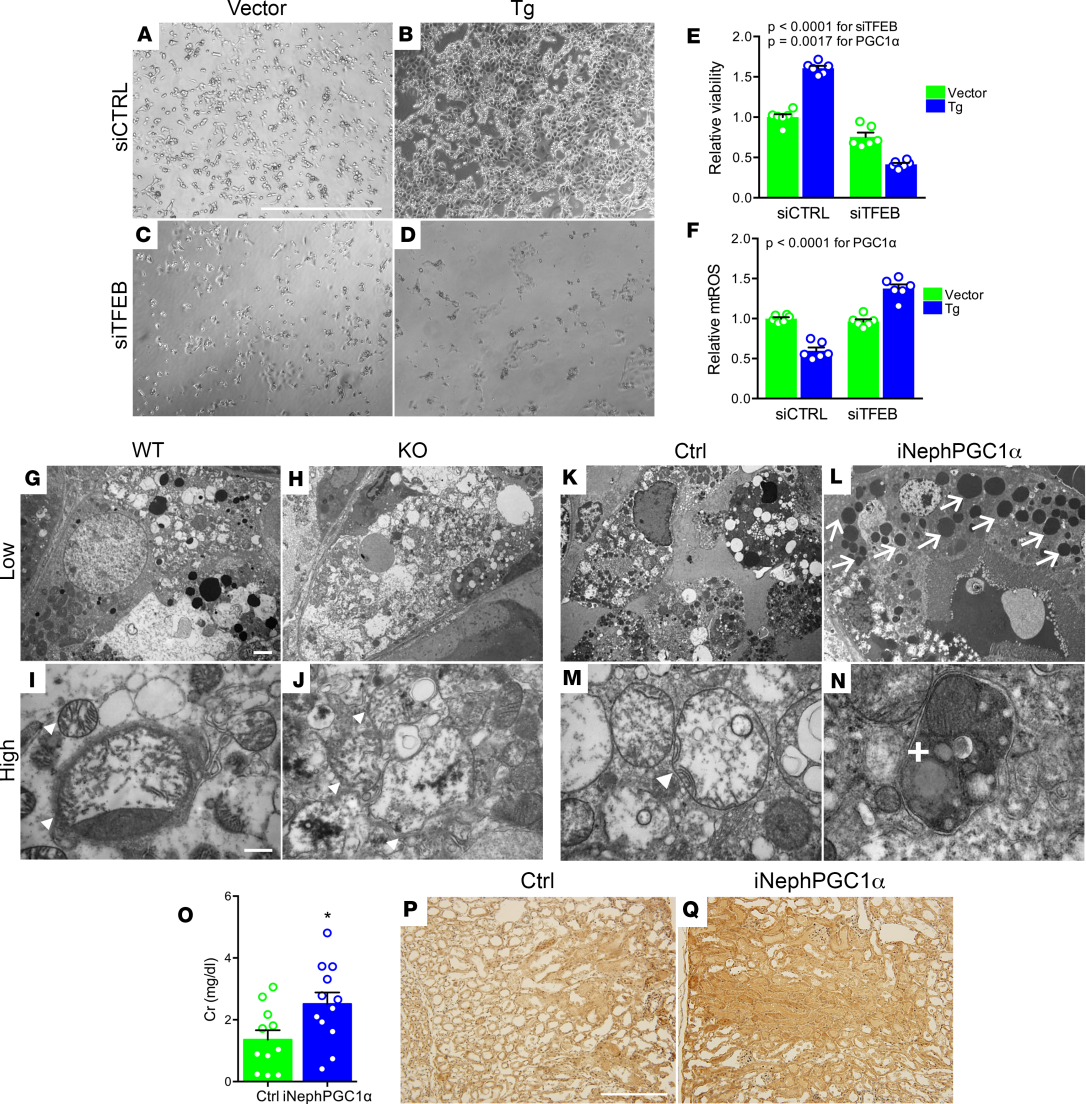
Lysosomes are pivotal for PGC1α-dependent protection against cisplatin. (**A–D**) Representative bright-field images (from *n* = 5/condition) of cisplatin-treated (10 μM, 24 hours) PGC1α Tg cells or vector controls TFEB siRNA knockdown (siCTRL or siTFEB, 50 μM, 72 hours). Scale bar: 1mm. (**E**) Viability via XTT assay in **A–D**. *n* = 6/condition. (**F**) Mitochondrial ROS (mtROS) via mitoSOX in (**A–D**). *n* = 6/condition. **E** and **F** analyzed by 2-way ANOVA. (**G–J**) Representative low- and high-power transmission electron microscopy (TEM, *n* = 3–5 mice/condition) of PGC1α-KO or WT proximal tubule 72 hours after cisplatin (20 mg/kg i.p.) showing swollen mitochondria (arrowheads). (**K–N**) Representative TEM of control or iNephPGC1α proximal tubule 72 hours after cisplatin (30 mg/kg i.p.) showing numerous electron-dense lysosomes (arrows), swollen mitochondria (arrowheads), and a swollen mitochondrion inside a membrane-bound structure consistent with mitophagy (plus sign). Scale bars: 2 μm (low power), 500 nm (high power). (**O**) Serum creatinine (Cr, mg/dl) in controls (*n* = 11) or iNephPGC1α (*n* = 12 mice) 72 hours after concurrent cisplatin (30 mg/kg i.p.) and chloroquine (10 mg/kg i.p.). (**P** and **Q**) Representative renal cortex staining (from *n* = 3–5 mice/condition) for 3-nitrotyrosine, a product of oxidative stress, in conditions indicated in (**O**). Scale bar: 200 μm. *P < 0.05 by Mann-Whitney *U* test.

This emerging concept suggested both a potentially novel tubular protection mechanism via TFEB and a way to reconcile how PGC1α enhances stress resistance, even as it adds potentially noxious mitochondrial mass. We therefore hypothesized that impairment of lysosomes would unmask a toxic effect of PGC1α related to mitochondrial injury. Cisplatin-treated PGC1α Tg cells were indeed more susceptible to TFEB depletion than cisplatin-treated controls (Figure 5, A–E). TFEB depletion fully reversed the suppressive effect of PGC1α on mitochondrial ROS (mtROS) induced by cisplatin (Figure 5F). Electron microscopy of cisplatin-treated PGC1α-KO mouse kidneys showed mitochondrial damage, whereas iNephPGC1α kidneys displayed abundant lysosomes and autolysosomes sequestering swollen mitochondria (Figure 5, G–N). Mirroring TFEB knockdown results, the lysosome inhibitor chloroquine now exacerbated cisplatin nephrotoxicity iNephPGC1α mice (Figure 5O), and cisplatin-treated iNephPGC1α mice displayed increased oxidative stress (Figure 5, P and Q). Finally, iNephPGC1α mice exhibited enlarged lysosomes and autolysosomes (Supplemental Figure 10).

## Discussion

The combination of loss- and gain-of-PGC1α — both in cells and in mice — provide consistent, powerful evidence that PGC1α in the renal tubular epithelium critically affects metabolic, cellular, and physiological responses to toxic injury. Together with published inflammatory and postischemic AKI models (6, 26), the data are notably concordant: starting with less PGC1α worsens unrelated forms of acute renal tubular injury, whereas starting with more PGC1α affords protection.

The present results mechanistically elucidate how PGC1α benefits stressed renal tubular epithelial cells without exposing those cells to the downside risks of noxious potentiators that emanate from injured mitochondria such as mitochondria-derived free radicals. In nonrenal models, diverse extramitochondrial PGC1α effectors have been implicated (27–29). In the case of neuronal PGC1α, these effectors include antioxidant enzymes that can detoxify mitochondrial free radicals (8). In contrast to these results outside of the kidney, renal tubular PGC1α defends mitochondrial energy extraction even during stress (6, 7, 26, 30, 31) and without the induction of antioxidant enzymes (6). The gain-of-function mouse model employed herein increases renal tubular mitochondrial abundance before the onset of injury (6, 7), raising questions about how the renal tubule is spared from mitochondrial oxidants once cisplatin is administered (32). Our data propose that PGC1 acts via TFEB to accelerate mitochondrial turnover. During injury, this may enable efficient and safe disposal of damaged mitochondria, thereby limiting intracellular exposure to oxidants emanating from damaged mitochondria. Coupled with its mitochondrial biogenesis function, the net effect of PGC1α in the stressed renal tubule may be to shift the distribution of mitochondria toward healthier organelles with better energy extraction and less free radical production. The PGC1α pathway is so strongly reliant on TFEB and lysosomes that its effect during stress flips from protection to harm depending on these effectors’ status. This 2-fold action of PGC1 — lysosomes curtailing mitochondrial oxidants plus healthier mitochondria defending ATP production — may, thus, be central to its wide-ranging renoprotection.

We recently reported that PGC1α induces a biosynthetic pathway for cells to increase the coenzyme NAD^+^ (6, 21). As the chief electron acceptor from glycolysis and the tricarboxylic acid cycle, sole electron donor to complex I of the electron transport chain, and a requisite substrate for metabolism-modifying sirtuin enzymes, it is perhaps unsurprising that intracellular NAD^+^ levels have been reported to be rate-limiting for oxidative metabolism (33). For highly oxidative cells, this means that a reduction in NAD^+^ translates into less efficient mitochondrial function and, thus, less ATP. During AKI, renal NAD^+^ levels decline because PGC1α-dependent NAD^+^ biosynthesis falls and because NAD^+^ consumption rises due to stress-induced enzymes such as poly-ADP ribose polymerases (PARPs) (6, 34). Consistent with this body of results, administration of PGC1α-independent NAD^+^ precursor compounds ameliorates different forms of experimental AKI, as shown here and previously (6). The present results further show that NAD^+^ augmentation mimics PGC1α’s effects on mitophagy. While the exact mechanisms linking NAD^+^ to mitophagy need to be elucidated, the literature suggests 2 intriguing possibilities. First, as a substrate for sirtuins, NAD^+^ has been shown to promote PGC1α deacetylation, a posttranslational modification that increases PGC1α’s transcriptional coactivator function (35). Thus NAD^+^ is, on one hand, a downstream product of PGC1α, but on the other, it is an upstream activator of the protein. Alternatively, sirtuins can activate a coordinated transcriptional response to mitochondrial stress known as the mitochondrial unfolded protein response (UPR^mt^) (36). The UPR^mt^ may operate in parallel or in an overlapping fashion with mitophagy to restore healthy organellar function. Since the molecular links between UPR^mt^ and mitophagy have been elusive (37), future studies with cisplatin may yield new insights.

Multiple studies offer the apparent dueling perspectives that cisplatin increases autophagy and mitophagy, yet promotion of these processes counteracts its toxicity (38). The present data endorse a new synthesis. Mitophagy may be induced early after cisplatin but as an adaptive rather than toxic response. By a later time closer to overt kidney dysfunction, cisplatin may retard this process, thereby accelerating cell death (11). Our cellular results at 24 hours of exposure and in vivo results 72 hours after cisplatin suggest that cisplatin ultimately impedes mitophagy. Because mtKeima reports a shift to acidic pH in the environment of mitochondria, our results cannot distinguish whether mitophagy is blunted at the step of mitochondrial uptake into autophagosomes, after acidification as this unit matures into a degradative autolysosome, or both. Despite this limitation, the results support a model in which effective mitochondrial clearance is blunted following cisplatin. The late failure to maintain adaptive clearance mechanisms could be a proximate cause of cell death and account for the delayed rise in serum creatinine that characterizes the clinical syndrome of cisplatin nephrotoxicity. Detailed autophagy and mitophagy flux studies are needed to illuminate the kinetics of this process.

TFEB may be a new target in AKI. Monogenic lysosomal diseases illustrate how important safe waste disposal is for human renal health (39). Termed the Coordinated Lysosomal Expression And Regulation network (CLEAR network), TFEB’s gene targets link early steps of autophagy to lysosomal biogenesis to drive an integrated program for cellular waste removal (40, 41). We found that PGC1α is required for intact renal tubular TFEB expression, that more PGC1α further induces TFEB, and that TFEB is required for PGC1α-dependent tubular protection. In the liver, TFEB promotes PGC1α transcription by directly ligating its promoter and may, in turn, be upregulated by one of PGC1α’s canonical transcription factor partners, PPAR (24). Such reciprocal regulation between PGC1α and TFEB may also be present in the renal tubule. Consequently, therapeutic TFEB activation during AKI could help break a vicious cycle triggered by injury-induced suppression of PGC1α (26). Development of small molecule TFEB agonists should enable studies in genetic models and acquired forms of PGC1α deficiency. Careful investigation of TFEB agonists at multiple time points may also elucidate the time window in which lysosomes are most important to combat cisplatin’s toxic renal effects. Conversely, the exciting prospect that PGC1α could modulate chronic lysosomal diseases affecting the kidney should be tested.

In summary, the present results address a continuum of mitochondrial homeostasis in the injured kidney spanning the production and disposal of these organelles; identify blunted mitophagy as a potentially novel effect of cisplatin; define PGC1α as a determinant of renoprotection against cisplatin; and implicate TFEB as a new mediator of renal stress resistance. These results could have future impact on the treatment of common acute and rare renal diseases.

## Methods

### Creation of PGC1α-KO cell lines.

PGC1α-KO cells were created from an immortalized mouse inner medullary collecting duct cell line (mIMCD-3, ATCC). The CRISPR/Cas9 system used to knock out PGC1α was described by Ran et al. (42). LentiCRISPR v2 (Addgene plasmid no. 52961) was a gift from Feng Zhang (Broad Institute of Harvard and MIT, Cambridge, MA, USA) (43). The sgRNA sequence used to target the mouse PGC1α coding sequence was 5′-CCGCTCGGATTTCCTGGTCT-3′. Successfully transfected cells were selected using puromycin. Single cell colonies of selected cells were created using the clonal dilution method. Clones were validated by sequencing the region flanking the sgRNA target sequence and by biochemical assays, as described. A control cell line was created in parallel with a control sgRNA that was noncomplementary to any mouse genomic sequence.

### Creation of PGC1α Tg cell lines.

PGC1α Tg cells were created by transduction of mIMCD-3 cells with a third-generation lentiviral transfer plasmid expressing the mouse PGC1α open reading frame. Cells were cotransfected using the psPAX2 packaging plasmid and the pMD2.G envelope plasmid. pLenti CMV GFP Puro (658-5; Addgene plasmid no. 17448) was a gift from Eric Campeau and Paul Kaufman (University of Massachusetts Medical School, Worcester, MA, USA) (44). GFP-PGC1α (Addgene plasmid no. 4) was a gift from Bruce Spiegelman (Dana Farber Cancer Institute, Boston, MA, USA) (45). psPAX2 and pMD2.G (Addgene plasmid nos. 12260 and 12259, respectively) were gifts from Didier Trono (Ecole Polytechnique Federale de Lasuanne, Lausanne Switzerland). Lentiviral particles were created by transfection of HEK293T cells and subsequently concentrated using the Lenti-X concentrator (Clontech). Transfected cells were selected with the addition of puromycin to complete media at 1 μg/ml. Tg cells were validated by confirming PGC1α overexpression using quantitative PCR (qPCR). Tg control cells were created in parallel and contained an empty lentiviral expression vector; they were also maintained in puromycin-containing media.

### Viability and ATP assays.

Cell viability was assessed using an XTT assay (ATCC). ATP measurements were determined with a luminescent kit (Abcam, ab113849). mtROS in cells were determined by MitoSOX assay per manufacturer’s instructions (Thermo Fisher Scientific). Cells were transfected with siRNA targeting mouse TFEB or a negative control siRNA (Thermo Fisher Scientific) for 72 hours.

### Oxygen consumption experiments.

Cells were seeded in an XF 24-well cell culture plate (Seahorse Bioscience) at 4 × 10^4^ cells per well and grown in complete medium. Prior to oxygen consumption analysis, media was changed to Seahorse XF Base Medium (Seahorse Bioscience) supplemented with 10 mM glucose, and 1 mM pyruvate and adjusted to pH 7.4. Oxygen consumption rates (pmol/min) were assessed using an XF-24 Flux Analyzer (Seahorse Bioscience) at baseline, after the addition of the ATP synthase inhibitor oligomycin (1 μM), after the addition of the uncoupling agent 2,4-dinitrophenol (1 μM), and again after addition of the complex I inhibitors rotenone (0.5 μM) and antimycin A (0.5 μM).

### Western analysis.

Cell lysate preparation, gel electrophoresis, transfer, immunoblotting, detection, and image acquisition were performed as previously described (6, 26). Antibodies used were TFEB (Bethyl Labs, A303-673A) and p62 (MilliporeSigma, P0067).

### Mitophagy and lysosomal measurements.

Detailed methods for the measurement of mitophagy based on mtKeima have been described previously (19, 20). Briefly, fluorescence of mtKeima was imaged in 2 channels via 2 sequential excitations (458 nm, green; 561 nm, red) and using a 570–695 nm emission range by live confocal microscopy (Zeiss). A mitophagy index was calculated by determining the ratio between the area of the red (acidic) and green (basic) emission. For mitophagy index calculation in mtKeima mice, the average of 4 images from each tissue sample was taken, and the values were normalized to the average value seen in the controls, assigned the value of 1. In each experimental model, all imaging parameters remained the same for all data acquisition using Zen Zeiss software. Acidified lysosomes were labeled using LysoTracker Red DND-99 (Thermo Fisher Scientific) and imaged at an excitation/emission of 577/590 nm using live confocal microscopy.

### RNA sequencing and bioinformatics.

Poly(A)-enriched RNA was isolated from PGC1α-KO and Tg cells and their respective genotype controls (described above) treated with cisplatin 10 μM for 24 hours. Three replicates were collected per condition and checked for quality on denaturing agarose gel. Sequencing libraries were generated from double-stranded cDNA using the Illumina TruSeq kit according to the manufacturer’s instructions. Library quality was checked using the Agilent DNA High Sensitivity Chip and qPCR. High-quality libraries were then sequenced on an Illumina NextSeq 2000. To achieve comprehensive coverage for each sample, approximately 25 million to 30 million paired-end reads were generated. Raw results were passed through quality control steps and aligned to the mouse genome. Gene expression determinations were performed from aligned reads by counting unique reads. Read-count–based expression data were normalized by the voom method, which estimates the mean variance relationship of log-counts and assigns a weight to each observation prior to linear modeling. Normalized count data were compared between groups using a linear modeling approach by implementing the Limma R package to identify differentially expressed genes based on a multiple-test–corrected *P* value and fold change (46). To extract patterns in genes that were significantly altered (multiple test corrected *P* < 0.05 and absolute fold change > 2) in PGC1α-KO and Tg cells, SOM analysis was performed (26). For example, clusters 2 and 5 indicated gene sets oppositely regulated in the test conditions — either elevated in KO and depressed in Tg or depressed in KO and elevated in Tg. This gene set was then evaluated for overrepresentation of genes described in Settembre et al. (25) or against a curated database of genes involved in lysosomal biology (http://lysosome.unipg.it) by applying the Fisher’s exact test. Sequencing data have been uploaded at https://www.ncbi.nlm.nih.gov/geo/ under accession no. GSE126259.

### Mouse studies.

Tubule-specific PGC1α–conditionally overexpressing Tg (Pax8-rtTA x tetO-PGC1α, referred to as iNephPGC1α or Tg mouse), PGC1α-KO, and mtKeima mice have been previously described (6, 20, 26). Each parent strain was obtained from the Jackson Laboratory, where extensive details on strain background are available: Pax8-rtTA (stock no. 007176); tetO-PGC1α (stock no. 012387); PGC1α KO (stock no. 008597); and mtKeima (stock no. 028072). Experiments were performed on male mice ages 8–11 weeks using littermate controls by an operator blinded to genotype and randomized within each cage to vehicle vs. AKI model.

Cisplatin, NMN, and chloroquine were purchased from MilliporeSigma. All treatments administered to mice were given by i.p. injection. Cisplatin treatment was 20 mg/kg for PGC1α-KO and mtKeima mice and 30 mg/kg for iNephPGC1α Tg mice. These doses were based on previous literature (47) and informed by published guidance from the United States Food and Drug Administration (FDA) as described below. NMN (400 mg/kg) was given 24 hours and 1 hour prior to cisplatin, followed by a final dose at 24 hours after cisplatin treatment. Chloroquine (10 mg/kg) was given 1 hour prior to cisplatin and then every 24 hours until sacrifice (48). Serum and organs from all cisplatin-treated mice were collected and analyzed at 72 hours. Creatinine from mouse serum was measured using liquid chromatography–tandem mass spectrometry (LC/MS-MS) at the University of Alabama Birmingham O’Brien Core Center for Acute Kidney Injury Research (Birmingham, Alabama, USA) in a blinded fashion (NIH P30 DK079337).

A typical human dose of cisplatin is 60 mg/m^2^ i.v. per cycle. For a 60 kg individual with a height of 65 inches, this calculates to 100 mg (i.e., 1.67 mg/kg). Per guidance from the FDA (49), this human dose in mg/m^2^ can be scaled to mouse dose in mg/kg either by dividing the human dose by 3 (60 mg/m^2^ for human/3 = 20 mg/kg for mouse) or by converting the human dose into mg/kg and then multiplying by 12.3 (1.67 mg/kg for human × 12.3 = 20.5 mg/kg for mouse).

### qPCR.

Total RNA extraction and cDNA synthesis were performed as previously described (6). PCR reactions were performed in duplicate using QuantStudio 6 Flex Real-Time PCR System (Applied Biosystems). SYBR primers were designed using PrimerQuest Tool (Integrated DNA Technologies). Relative expression levels were determined using the comparative threshold method. Measured transcripts included genes involved in PGC1α-dependent mitochondrial biogenesis (TFAM, transcription factor A mitochondrial; ATP5O, ATP synthase subunit O mitochondrial; and NDUFS1, NAD-ubiquinone oxidoreductase 75 kDa subunit mitochondrial); as well as genes involved in PGC1α-dependent NAD^+^ biosynthesis (IDO2, indole dioxygenase 2; AFMID, arylformamidase; QPRT, quinolinate phosphoribosyl transferase; NADSYN1, NAD synthetase 1; and NAMPT, nicotinamide phosphoribosyltransferase) (6, 9).

### Structured illumination microscopy.

Paraffin embedded kidney tissue was sectioned at 4–6 μm and mounted on silanized ([3-Aminopropyl] trimethoxysilane, MilliporeSigma) cover glass (limited working distances for super resolution objectives necessitates mounting the tissue directly to the coverslip). Antigen retrieval was carried out by incubating the sections in 0.1 M citrate buffer at high temperature (115°C) and high pressure using a pressure cooker. Tissues were blocked with normal donkey serum and stained with anti-LAMP2 (Abcam, clone GL2A7) and anti-LC3 (NanoTools, clone 5F10, Biotinylated) antibodies. Donkey anti-Rat Cy3 secondary (Jackson ImmunoResearch, catalog 712-166-153) was used to detect anti-LAMP2, and Streptavidin DyLight 488 (Invitrogen, catalog 21832) was used to detect anti-LC3. Nuclei were stained with DAPI (MilliporeSigma). Tissues were imaged on a N-SIM microscope (Nikon structured illumination microscope) using a 100× TIRF super-resolution objective and taken as a Z-stack at 0.12 μm/Z-step. The resulting Z-stack was processed in Imaris software (Bitplane) to generate 3-dimensional surface renderings and graphs of rendered structures. SIM imaging and image processing was performed in part through the use of the Vanderbilt Cell Imaging Shared Resource and Nikon Center of Excellence (supported by NIH grants CA68485, DK20593, DK58404, DK59637 and EY08126).

### Electron microscopy.

The complete method is previously described (32, 50). Briefly, kidneys were fixed with 2.5% glutaraldehyde in 0.1 M cacodylate buffer (pH 7.4) and cut in 1-μm sections in both sagittal and transverse planes for image analysis. After drying the sections, slides were stained at 65°C for 20 minutes in 0.1% Toluidine blue in 1% sodium borate, cooled to room temperature, washed in distilled water, cleaned in xylene, and mounted in Permount sections for light microscopy. Subsequent ultrathin sections (0.5 μm) were examined by transmission electron microscopy (JEOL 1011, JEOL Corp.) with Orca-HR Digital Camera (Hamamatsu Corp.) and Advanced Microscopy Technique Corporation image capture system.

### Nitrotyrosine immunohistochemistry.

Tyrosine nitration was measured in 5 micrometer kidney sections prepared from formalin-fixed paraffin-embedded tissues. Sections were incubated overnight at 4oC in 1:1500 dilution of rabbit polyclonal anti-nitrotyrosine antibody (A-21285 IgG2b; Thermo Fisher). Slides were then rinsed in PBS and developed using ImmPRESS HRP anti-rabbit IgG (peroxidase) polymer detection kit (Vector Laboratories) per the manufacturer’s directions.

### Statistics.

For all studies, nonparametric tests were used to compare continuous variables (Mann-Whitney *U* test or Kruskal Wallis if >2 groups) unless otherwise noted. Two-way ANOVA was employed for grouped analyses, with *P* values corrected for multiple comparisons as appropriate. Data are presented as mean ± SEM unless otherwise specified. Power for mouse studies was guided by the following calculation from pilot studies of cisplatin nephrotoxicity: serum creatinine of 0.8 ± 0.3 mg/dl in WT mice vs. 1.5 ± 0.5 mg/dl in PGC1α-KO mice requires 7 mice per group for >90% power. Mouse AKI models were performed by an operator blinded to genotype and with random assignment to control vs. injury condition. Serum creatinine was measured by a core service indicated above that was blinded to experimental conditions. Histopathology and ultrastructure were evaluated by operators blinded to the condition. Results were prepared using Graphpad Prism Version 7. Two-tailed *P* < 0.05 were considered significant. *P < 0.05, **P < 0.01, ***P < 0.001, ****P < 0.0001 unless otherwise indicated.

### Study approval.

Mouse studies were conducted with approval by the IACUC at Beth Israel Deaconess Medical Center and the National Heart, Lung and Blood Institute.

## Author contributions

MRL conducted cellular studies focused on autophagy and mitophagy measurements. MTT conducted RNA interference studies on TFEB and mouse studies, with assistance from XC. KMR developed and characterized stable KO and Tg cells for PGC1α with help from VR. ZKZ, DB, and IES conducted histopathological studies on mouse tissues. SSB and MKB analyzed RNA sequencing results. NS, IIR, and TF studied mtKeima Tg mice. CRB and KT conducted structured illumination microscopy studies on kidneys from PGCα genetic models treated with cisplatin. MTT, MRL, KMR, and SMP prepared the manuscript with input from all authors.

## Supplementary Material

Supplemental data

## Version 1 03/14/2019

In-Press Preview

## Version 2 04/18/2019

Electronic publication

## Version 3 08/04/2020

Figure 5 was corrected.
