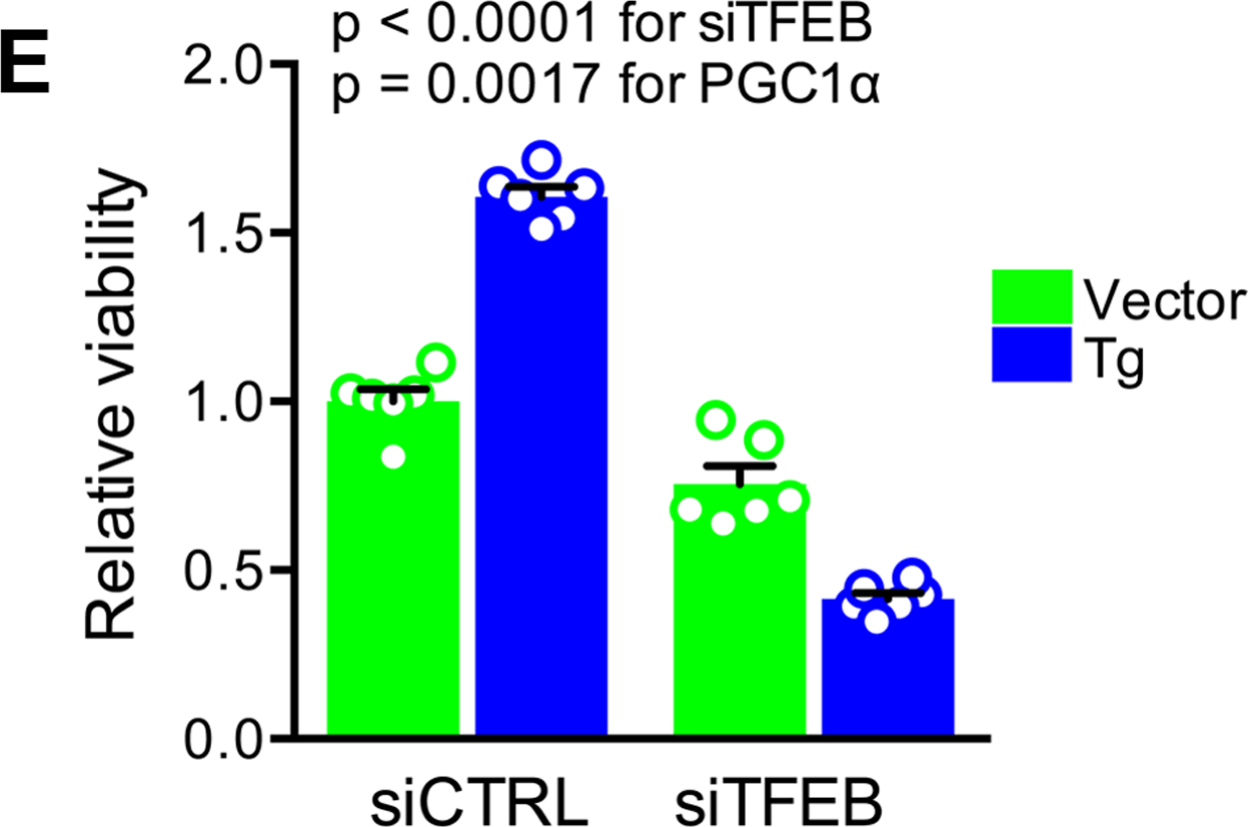# TFEB-driven lysosomal biogenesis is pivotal for PGC1﻿α﻿﻿-dependent renal stress resistance

**DOI:** 10.1172/jci.insight.142898

**Published:** 2020-08-06

**Authors:** Matthew R. Lynch, Mei T. Tran, Kenneth M. Ralto, Zsuzsanna K. Zsengeller, Vinod Raman, Swati S. Bhasin, Nuo Sun, Xiuying Chen, Daniel Brown, Ilsa I. Rovira, Kensei Taguchi, Craig R. Brooks, Isaac E. Stillman, Manoj K. Bhasin, Toren Finkel, Samir M. Parikh

Original citation: *JCI Insight*. 2019;4(8):e126749. https://doi.org/10.1172/jci.insight.126749

Citation for this erratum: *JCI Insight*. 2020;5(15):e142898. https://doi.org/10.1172/jci.insight.142898

When the article was originally published, an incorrect panel was included in Figure 5E. The correct figure part is below. The HTML and PDF files have been updated online.

JCI Insight regrets the error.

## Figures and Tables

**Figure 5:**