# Supplementary material for: TFEB-driven lysosomal biogenesis is pivotal for PGC1α-dependent renal stress resistance
Source: JCI Insight. 2019 Apr 18;4(8):e126749. doi: 10.1172/jci.insight.126749 (PMC6538327; doi:10.1172/jci.insight.126749)

Supplemental Data for “TFEB-driven lysosomal biogenesis is pivotal for PGC1α-dependent renal stress resistance”

Supplemental Figure 1: Generation of a stable PGC1α KO cell line. (A) PGC1α expression was measured using quantitative real-time PCR in 12 clonal population of cells which had undergone CRISPR-mediated PGC1α knockout. All values are normalized to control cells transfected with Cas9 and a control sgRNA sequence. (B) At the same confluency and time since last media change, the media in the PGC1α KO cells was more acidic than control cells, as visualized with the phenol red pH indicator in the media. (C) Genomic sequencing of control cells and cells from PGC1α KO clone A2, demonstrating that the region around the sgRNA target site has been mutated.

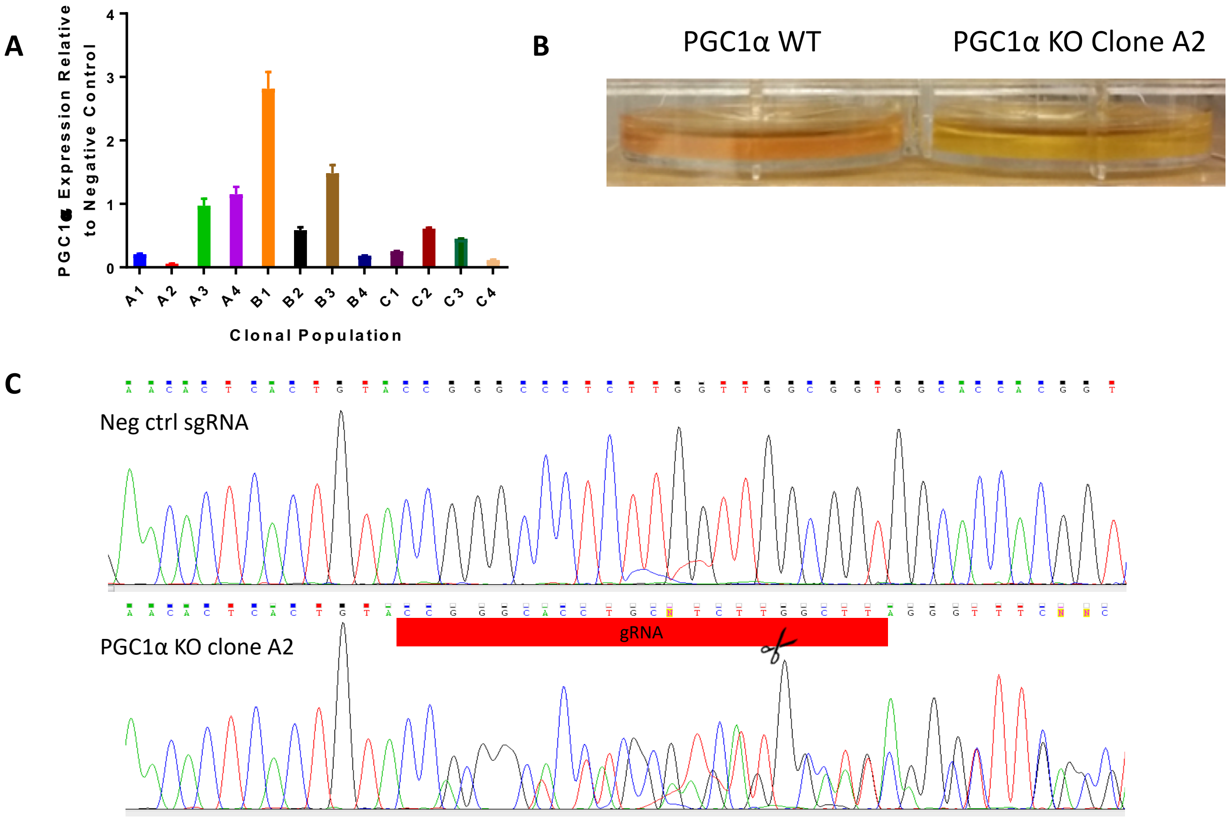

Supplemental Figure 2: Development of a stable PGC1α transgenic cell line. (A) Map showing the lentiviral transfer plasmid used for transduction of mIMCD-3 cells to create a stable PGC1α transgenic cell line (insert in gray). (B, C) Total NAD (a.u.) in control vector vs. PGC1α transgenic (Tg) cells at baseline (B) and following cisplatin (C, 10μM, 24h).

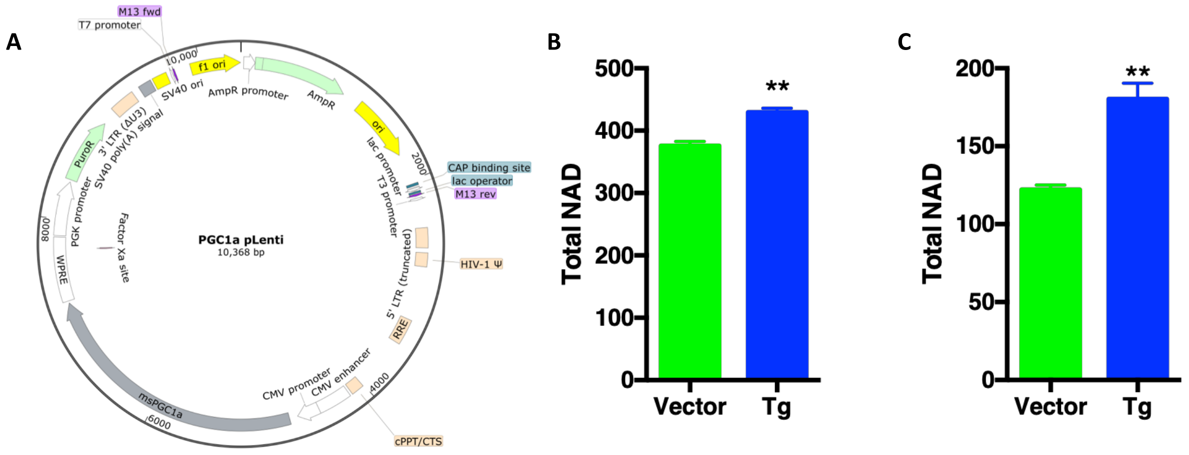

Supplemental Figure 3. PGC1α-dependent modulation of respiration. Cellular oxygen consumption rate (OCR) was measured using the Mito Stress Kit with a Seahorse XF24 analyzer. Arrows correspond to the sequential addition from left to right of oligomycin, the mitochondrial uncoupler FCCP, and Rotenone+Antimycin. (A, B) $P < 0.0001$ for genotype by two-way ANOVA comparing PGC1α KO cells to controls (A). $P < 0.0001$ for genotype by two-way ANOVA comparing PGC1α transgenic cells to vector controls (B).

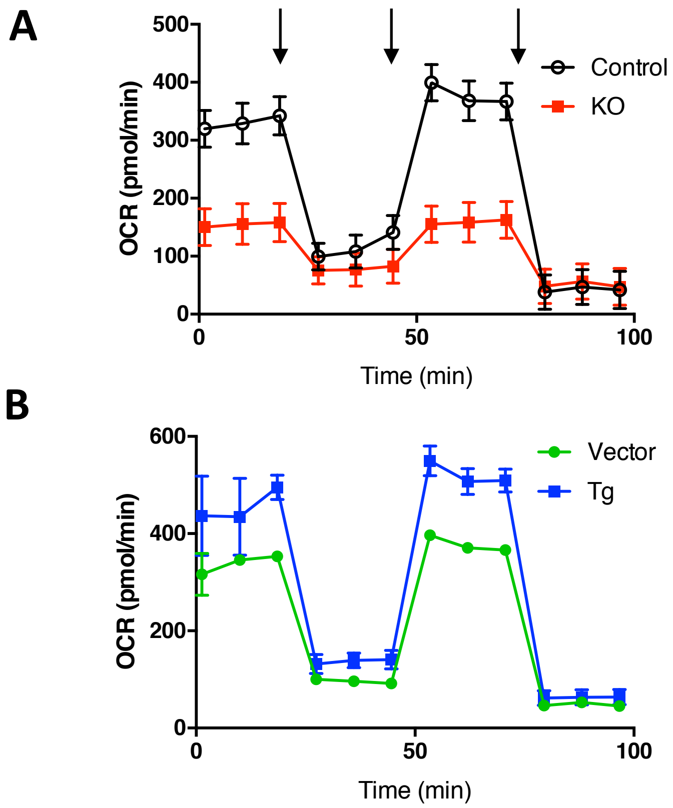

Supplemental Figure 4: **Cisplatin is dose-dependently cytotoxic to renal tubular cells and nephrotoxic in mice**. (A) Dose series for cisplatin applied to IMCD3 cells for 24h with cell viability measured by XTT assay. Red line indicates idealized inhibitor response and r^2 indicates goodness of fit with experimental results. N=3/group. (B) Renal function 72h after intraperitoneal cisplatin injection at the indicated dose in 12-week old male mice. N=3/group.

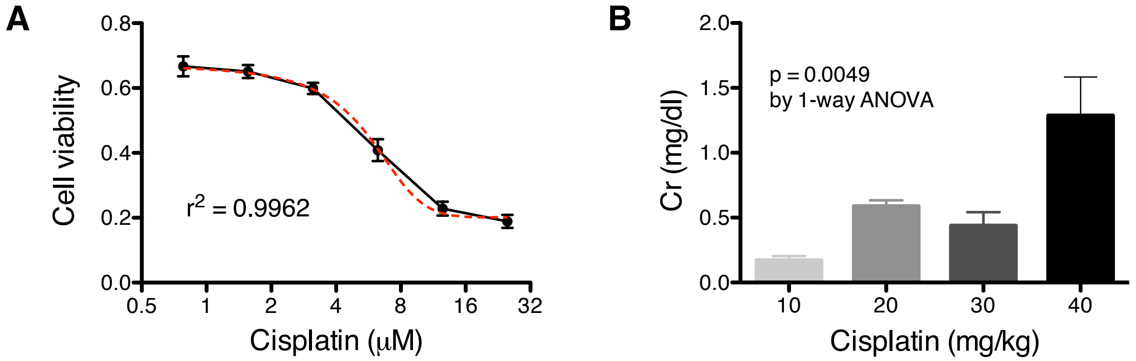

Supplemental Figure 5: PGC1α gene status is a determinant of renal tubule injury following cisplatin. (A) Semi-quantitative PCR for KIM-1 (kidney injury molecule 1) performed on kidneys from PGC1α KO or wildtype (WT) littermates treated with cisplatin (20 mg/kg, 72h prior to measurement). Results normalized to KIM-1 expression in WT kidneys 72h after vehicle treatment (dashed line). (B) Semi-quantitative PCR for KIM-1 performed on kidneys from iNephPGC1α mice (Tg) or littermate controls (Ctrl) treated with cisplatin (30 mg/kg, 72h prior to measurement). Results normalized to KIM-1 expression in control kidneys 72h after vehicle treatment (dashed line). P-values for PGC1α genotype by two-way ANOVA.

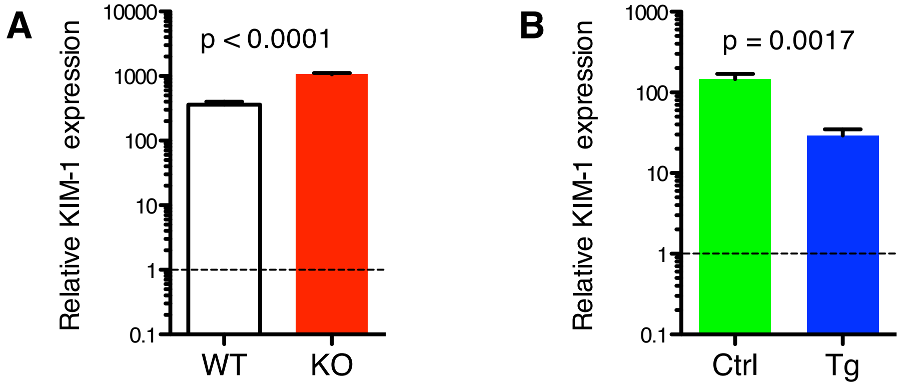

Supplemental Figure 6: Effect of PGC1α gene status on cisplatin-induced changes in autophagic marker p62. (A) Representative Western analysis for p62 of PGC1α KO vs. control cells after cisplatin (10μM, 12h). (B) Representative Western analysis for p62 of PGC1α transgenic cells (Tg) vs. vector controls. (C, D) Densitometry results for A, B. Analysis done using two-way ANOVA with multiple comparisons. NS = non-significant, *p < 0.05.

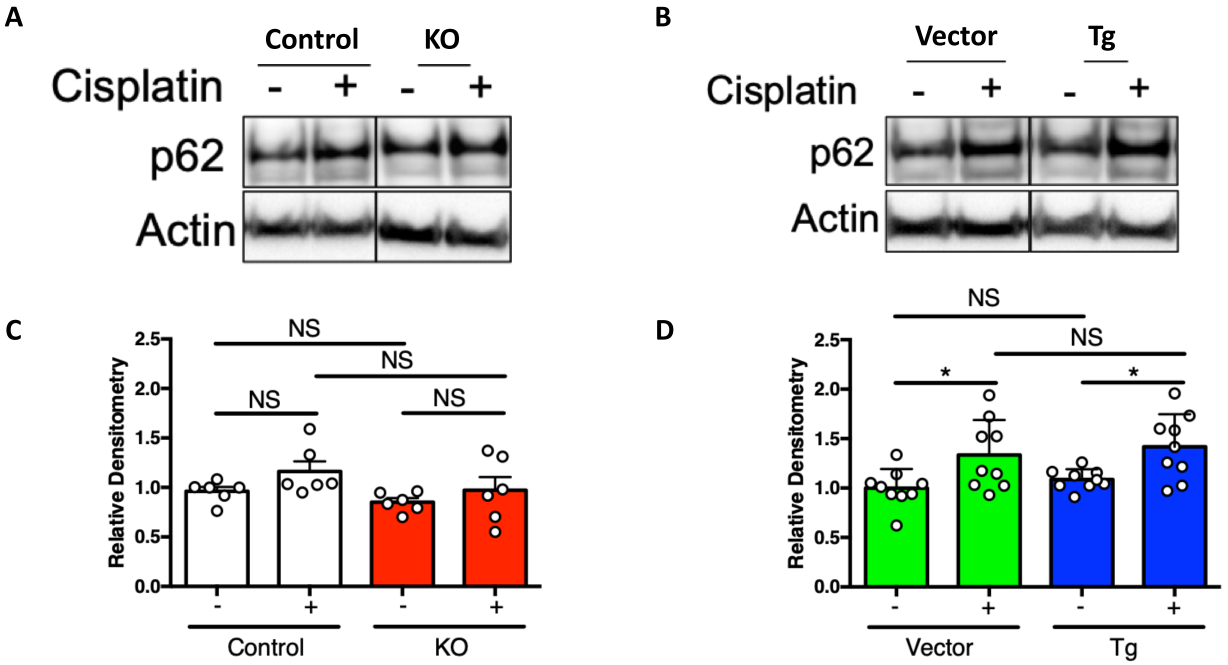

Supplemental Figure 7: Effect of PGC1α gene status on cisplatin-induced changes in mitophagic marker PINK1. (A) Semi-quantitative PCR for PINK1 (PTEN-induced kinase protein 1) in PGC1α KO vs. control cells after vehicle or cisplatin (10μM, 24h). (B) Semi-quantitative PCR for PINK1 in PGC1α transgenic (Tg) cells vs. vector control cells after vehicle or cisplatin (10μM, 24h). Analysis done using two-way ANOVA with multiple comparisons. *$p < 0.05$.

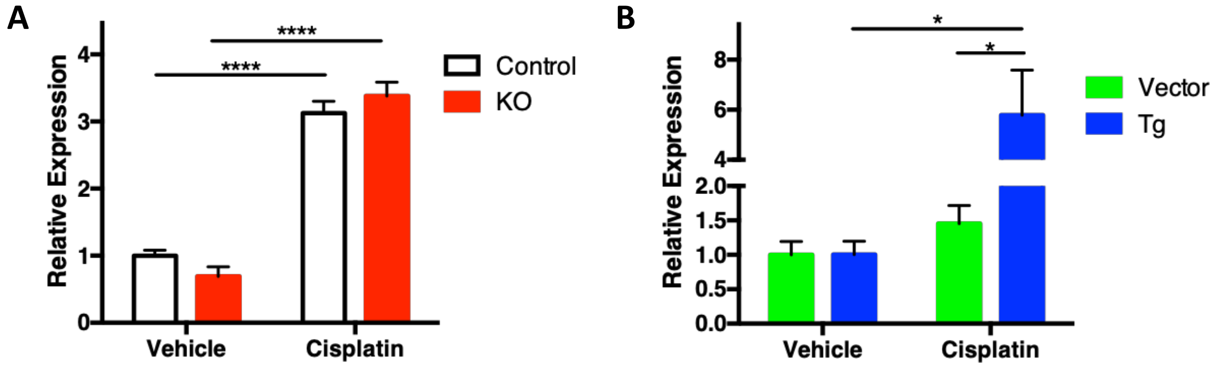

Supplemental Figure 8: Cisplatin reduces mitophagy in renal tubular cells. (A) Representative images in green channel (pH = 8.0) and red channel (pH = 4.0) of renal tubular cells treated with cisplatin (10μM, 24h). (B) Normalized quantification of A, n = 20 per condition. ****p < 0.0001 by Mann-Whitney.

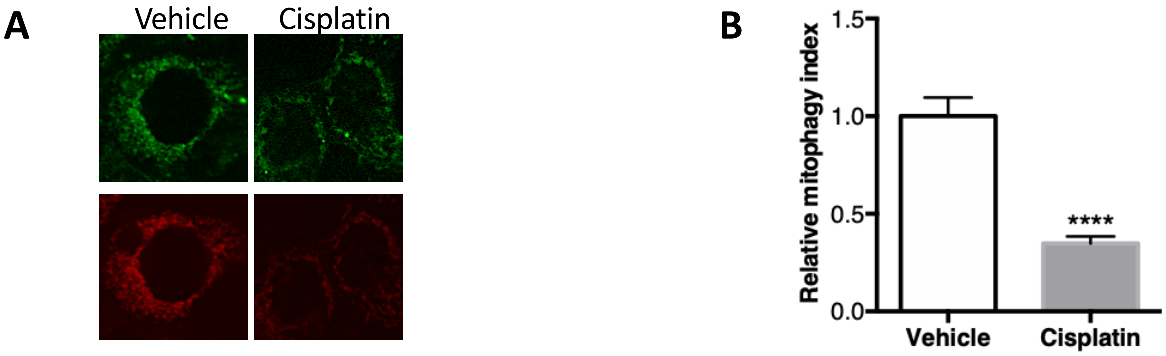

Supplemental Figure 9: TFEB-dependent genes and lysosomal genes are over-represented among PGC1α-oppositely regulated transcripts during cisplatin exposure. Transcripts from clusters 2 and 5—i.e. genes oppositely regulated by loss- vs. gain-of-PGC1α during cisplatin exposure (10μM, 24h)—were evaluated for over-representation of genes implicated by Settembre, et al., as TFEB-dependent gene products (1) or included in a curated database for lysosomal biology. P-value by Kolmogorov-Smirnov test.

Gene Set	P-value	Overlapping Genes
TFEB associated (Settembre, et al. Science 2011)	0.006	Map1lc3a, App, Eif2ak3, Eva1a, Wipi1
http://lysosome.unipg.it	0.012	Gm2a, Ctsc, Ap1m2, Manaba, Ap4s1, Lrp2, Napsa, Gnb4, Tfeb, Tspan8, Ldlr, Serinc2, Gusb, Lrp1, Myo7a, Ada, Atp6v0a4

[1] Settembre C, Di Malta C, Polito VA, Garcia Arencibia M, Vetrini F, Erdin S, et al. TFEB links autophagy to lysosomal biogenesis. *Science.* 2011;332(6036):1429-33.

Supplemental Figure 10: iNephPGC1a mice have larger lysosomes and autolysosomes. Ctrl or iNephPGC1a mice were treated with cisplatin (30 mg/kg), cisplatin + chloroquine (10 mg/kg) or vehicle. Kidneys were harvested 72 h later and sections were stained for the lysosomal protein LAMP2 (red), the autophagosome protein LC3 (green), and nuclei with DAPI (blue), then imaged using structured illumination microscopy. The resulting images of cortical tubules were processed in Imaris software to generate 3D surface renderings of the organelles, which were then graphed by volume and fluorescence intensity. White dashed boxes are areas enlarged in the lower panels. HM = high-magnification. Scale bar = 3 μm.

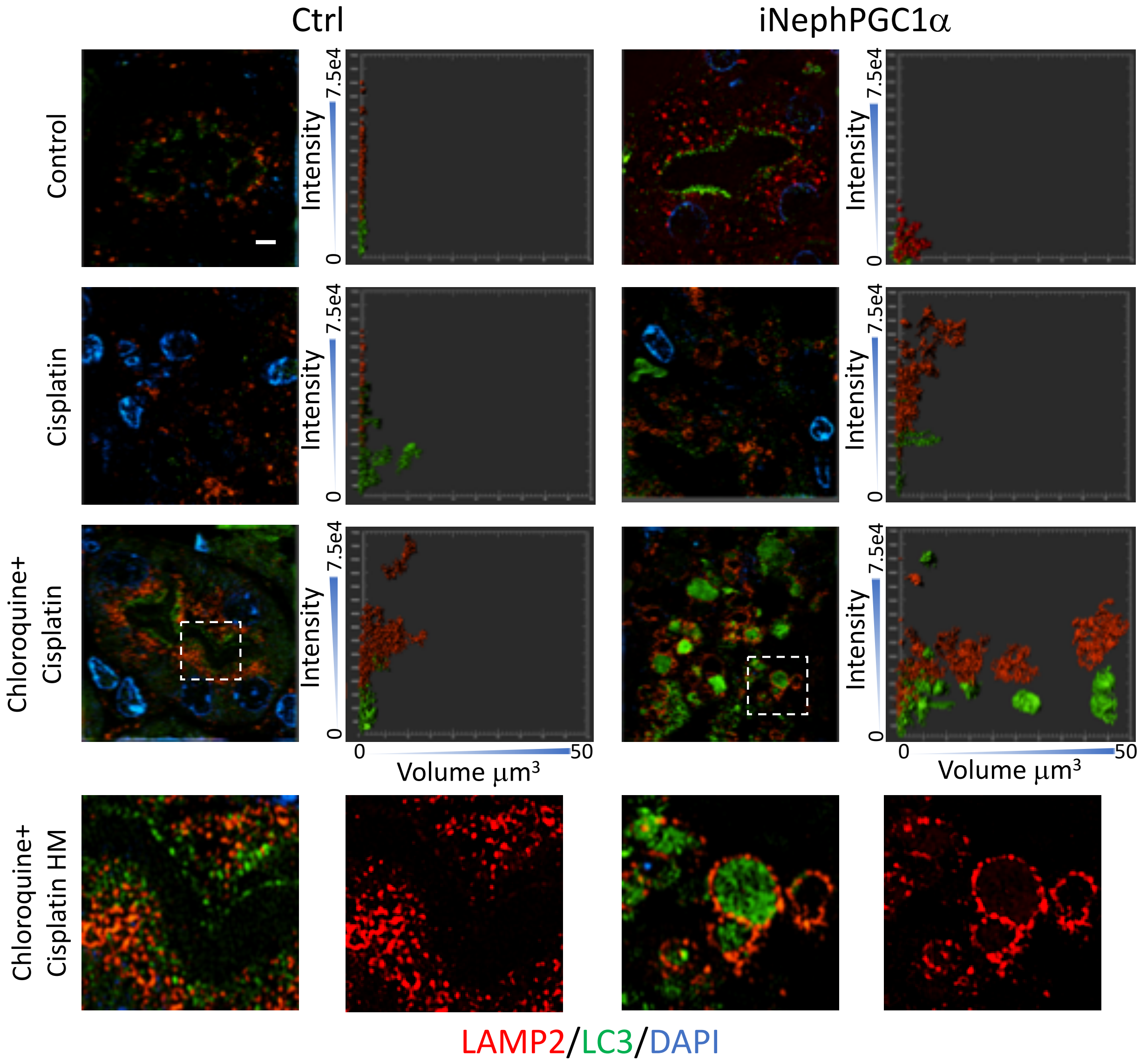

Supplement: Supplemental data [file jciinsight-4-126749-s006.pdf]
